# Identification and functional analysis of a three-miRNA ceRNA network in hypertrophic scars

**DOI:** 10.1186/s12967-021-03091-y

**Published:** 2021-10-29

**Authors:** Zewei Zhang, Xin Huang, Jiahao Yang, Shuchen Gu, Yixuan Zhao, Yunhan Liu, Yimin Khoong, Shuqi Wang, Shenying Luo, Tao Zan, Guangshuai Li

**Affiliations:** 1 Department of Plastic and Reconstructive Surgery, The First Affiliated Hospital of Zhengzhou University, Zhengzhou, 450000 China; 2 Department of Plastic and Reconstructive Surgery, Shanghai Ninth People’s Hospital, Shanghai Jiaotong University School of Medicine, Shanghai, 200001 China; 3 Department of Orthopedic, The First Affiliated Hospital of Zhengzhou University, Zhengzhou, 450000 China

**Keywords:** Hypertrophic scar, miR-422a, miR-2116-3p, miR-3187-3p, ceRNA network, Ubiquitination

## Abstract

**Background:**

Hypertrophic scar (HTS) is a fibrotic disorder of skins and may have repercussions on the appearance as well as functions of patients. Recent studies related have shown that competitive endogenous RNA (ceRNA) networks centering around miRNAs may play an influential role in HTS formation. This study aimed to construct and validate a three-miRNA (miR-422a, miR-2116-3p, and miR-3187-3p) ceRNA network, and explore its potential functions.

**Methods:**

Quantitative real‑time PCR (qRT‑PCR) was used to compare expression levels of miRNAs, lncRNAs, and genes between HTS and normal skin. Target lncRNAs and genes of each miRNA were predicted using starBase as well as TargetScan database to construct a distinct ceRNA network; overlapping target lncRNAs and genes of the three miRNAs were utilized to develop a three-miRNA ceRNA network. For every network, protein–protein interaction (PPI) network analysis was performed to identify its hub genes. For each network and its hub genes, Gene Oncology (GO) and Kyoto Encyclopedia of Genes and Genomes (KEGG) analysis were conducted to explore their possible functions.

**Results:**

MiR-422a, miR-2116-3p, and miR-3187-3p were all downregulated in HTS tissues and fibroblasts. MiR-422a-based ceRNA network consisted of 101 lncRNAs with 133 genes; miR-2116-3p-centered ceRNA network comprised 85 lncRNAs and 978 genes; miR-3187-3p-derived ceRNA network encompassed 84 lncRNAs as well as 1128 genes. The three-miRNA ceRNA network included 2 lncRNAs with 9 genes, where MAPK1, FOSL2, ABI2, KPNA6, CBL, lncRNA-KCNQ1OT1, and lncRNA-EBLN3P were upregulated. According to GO and KEGG analysis, these networks were consistently related to ubiquitination. Three ubiquitination-related genes (CBL, SMURF2, and USP4) were upregulated and negatively correlated with the expression levels of the three miRNAs in HTS tissues.

**Conclusions:**

This study identified a three-miRNA ceRNA network, which might take part in HTS formation and correlate with ubiquitination.

## Background

Hypertrophic scar (HTS) is a fibro-proliferative disease, charactered by excessive proliferation of fibroblasts, activation of myofibroblasts, and deposition of extracellular matrix (ECM). This disorder has the potential to cause disfiguration and dysfunction in facial areas and joints, which may take a heavy toll on the physical and psychological condition of patients. Despite increasing strategies towards this challenge, in an effort to seek more satisfactory management, the pathogenesis underlying HTS formation remains to be elucidated [1].

MicroRNAs (miRNAs) can be competitively sponged by mRNAs as well as long non-coding RNAs (lncRNAs), and these can form competitive endogenous RNA (ceRNA) networks, which have been reported to regulate multiple biological processes to engage in the pathogenesis of many diseases including HTS [2, 3]. For example, miR-422a has been shown to have the potential to inhibit cell proliferation in glioblastoma [4] and fibrotic genes in liver fibrosis [5]; miR-2116-3p has proven to hinder the proliferation of breast cancer cells [6]; miR-3187-3p has been reported to impede fibroblast proliferation [7]. Furthermore, in a preliminary microarray test, miR-422a, miR-2116-3p, and miR-3187-3p have been discovered to be downregulated in HTS tissues (fold change > 2 and P < 0.05) [8]. These studies have suggested that the three miRNAs may correlate with HTS formation and contribute positively to alleviating HTS. However, their expression patterns remain validated in tissues as well as fibroblasts, and their roles in HTS pathogenesis need to be answered.

A single lncRNA or mRNA tends to bear multiple binding sites for miRNAs, and can simultaneously sponge several miRNAs to constitute a sophisticated ceRNA network. This one-to-many pattern plays a crucial role in a range of fibrotic disorders including HTS. In identical tissues from patients suffering from HTS, dysregulated expression profiles of lncRNAs, miRNAs, and mRNAs have been observed to coexist and correlate [9]. Moreover, miR-23b-3p and miR-27b-3p have been confirmed to promote atrial fibrosis by targeting two distinct sites of transforming growth factor (TGF)-β1 receptor 3 (TGFBR3) [10]. In addition, miR-760 and miR-215-3p have been demonstrated to be competitively sponged by lncRNA NR_003923 and IL22RA1, promoting TGF-β-induced proliferation, migration, and autophagy of fibroblasts [11]. Nevertheless, it needs to be further investigated whether miR-422a, miR-2116-3p, and miR-3187-3p can be concurrently sponged by ceRNAs, contributing to the pathogenesis of fibrosis of many organs including skins.

**Fig. 1:**
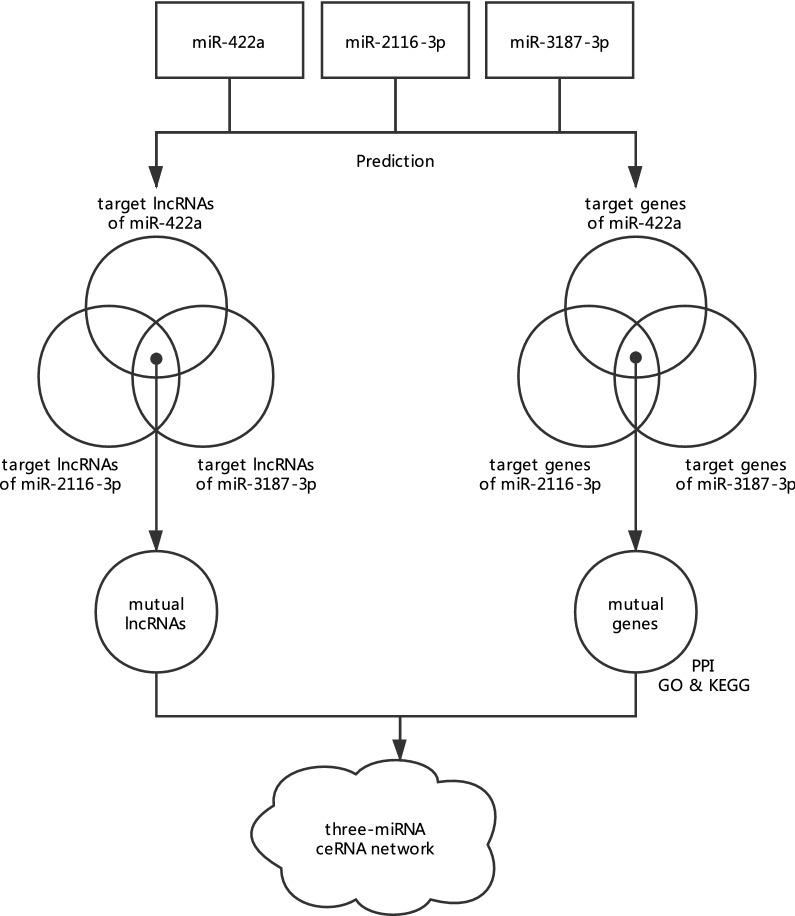
Flow diagram of ceRNA network construction. The three-miRNA (miR-422a, miR-2116-3p, and miR-3187-3p) ceRNA network was constructed by mutual lncRNAs as well as genes of the three miRNAs, and the common genes with their interacting proteins (predicted by NetworkAnalyst) were analyzed through PPI, GO, and KEGG analysis. *PPI* protein–protein interaction, *GO* Gene Oncology, *KEGG* Kyoto Encyclopedia of Genes and Genomes

In this study, we aimed to determine both expression profiles and possible functions of a three-miRNA (miR-422a, miR-2116-3p, and miR-3187-3p) ceRNA network in HTS. Therefore, we detected expression levels of these three miRNAs in HTS tissue samples and HTS-derived fibroblasts. In order to explore their roles in the pathogenesis of HTS, we predicted both target lncRNAs as well as genes and constructed ceRNA networks for each of the three miRNAs. These networks were subject to protein–protein interaction (PPI) network analysis to detect hub genes. GO and KEGG enrichment analysis was utilized to explore potential functions of both the networks and hub genes. Furthermore, we performed integrated bioinformatics analysis of miR-422a, miR-2116-3p, and miR-3187-3p to establish a comprehensive ceRNA network (Fig. 1), whose roles and expression patterns were analyzed through functional enrichment analysis and quantitative real-time PCR (qRT-PCR).

## Methods

### Tissue collection and cell culture

HTS tissues and normal skin samples were collected from 10 patients, who were informed about the aim as well as procedure of the study, signed written informed consent, and underwent HTS excision. Primary fibroblasts were isolated from HTS as well as normal skin, and cultured as previously described [12]. Briefly, tissues were minced and then placed in collagenase type IV (0.2 mg/ml, Roche) solution at 37 °C for 6 h. After filtering and centrifuging, fibroblasts were cultured in DMEM (Gibco, USA) supplemented with 10% fetal bovine serum (Gibco, USA) as well as 1% penicillin/streptomycin (Gibco, USA) in 5% CO_2_ at 37 °C. Fibroblasts between passage 3 and passage 9 were utilized for experiments. The study protocol was approved by the Medical Ethics Committee of The First Affiliated Hospital of Zhengzhou University and Shanghai Ninth People’s Hospital, Shanghai Jiaotong University School of Medicine.

### Quantitative real-time PCR (qRT-PCR)

Total RNA and miRNA were extracted form tissues and fibroblasts through TRIzol Reagent (Invitrigen, USA). 2 ng of total RNA and miRNA was subject to cDNA synthesis. Expression levels of protein-coding genes and miRNAs were normalized to those of GAPDH and U6, respectively. Primer sequences used in this research were listed as follows: GAPDH: 5’-GGGAAGGTGAAGGTCGGAGT-3’ (forward) and 5’-GGGGTCATTGATGGCAACA-3’ (reverse); KCNQ1OT1: 5’-GGCTTCTCGTTCAGGTGACA-3' (forward) and 5'-TGGTACATGGGCAGGGGATA-3' (reverse); EBLN3P: 5'-AATTTCGGGAGCTGTGACCC-3' (forward) and 5'-CAAAAGACTGCTGCCCAGTG-3' (reverse); ABI2: 5'-ACATGAAGATGGGTGGGCTG-3' (forward) and 5'-CTCCCACTGCTGCTGTAAGT-3' (reverse); FOSL2: 5'-AAACACCCTGTTTCCTCTCCG-3' (forward) and 5'-ATCTACCCGGAATTTCTGCTG-3' (reverse); MAPK1: 5'-CGAGTGACGAGCCCATCG-3' (forward) and 5'-GATGTCTGAGCACGTCCAGT-3' (reverse); CBL: 5'-ACGTGAAGAAGAGCTCTGGG-3' (forward) and 5'-ACAACCGCACCACCTTGTC-3' (reverse); KPNA6: 5'-TACTGAAAGCTGCCGCTGAA-3' (forward) and 5'-GCTCGCCATGGTCTCCATC-3' (reverse); SMURF2: 5'-CAGAGGACAACGCAACAAGG-3' (forward) and 5'-GTTGCTAAGATCCCTGGGCA-3' (reverse); USP4: 5'-GGTACGGCTGTGTAGAAGGC-3' (forward) and 5'-TCTCGATGGTTGCAATGGTG-3' (reverse); U6: 5’-CGCTAGCACATATCGGCTA-3’ (forward) and 5’-TTCTGCGACGAATTTGTCAT-3’ (reverse); miR-422a: 5’-GCATACCGCTATGCCTAATGGTG-3’ (forward) and 5’-GTGCAGGAGGTTCCGGT-3’ (reverse); miR-2116-3p: 5’-AATCCTATGCCAAGAACTCCC-3’ (forward) and 5’- CTCTACAGCTATATTGCCAGCCA-3’ (reverse); miR-3187-3p: 5’-GCCGAGTTGGCCATGGGGCTG-3’ (forward) and 5’- CTCAACTGGTGTCGTGGA-3’ (reverse).

### Construction of ceRNA networks

For each of miR-422a, miR-2116-3p, and miR-3187-3p, its target lncRNAs were predicted using starBase (http://starbase.sysu.edu.cn/); its target genes were forecasted by overlapping results from starBase as well as TargetScan (http://www.targetscan.org). Target lncRNAs and genes of every miRNA were utilized to develop its ceRNA network; overlapping target lncRNAs and genes of the three miRNAs were used to build up a three-miRNA ceRNA network. These networks were constructed through Cytoscape (version 3.7.0, http://www.cytoscape.org/).

### PPI network analysis

For target genes of every miRNA, a single PPI network was established using the Search Tool for the Retrieval of Interacting Genes (STRING) database (https:// string-db.org) and visualized through Cytoscape. The Cytoscape plugin, cytoHubba, was utilized to analyze each PPI network in an effort to identify its hub genes, whose ranks were calculated according to the built-in maximal clique centrality (MCC) algorithm. For genes in the three-miRNA ceRNA network, the PPI network was developed using interacting proteins, which were predicted to interact with the mutual genes by NetworkAnalyst (http://www.networkanalyst.ca/).

### GO and KEGG enrichment analysis

Using DAVID database (https://david.ncifcrf.gov/summary.jsp), Gene Ontology (GO) as well as Kyoto Encyclopedia of Genes and Genomes (KEGG) functional enrichment analysis was used to explore functions of genes in the ceRNA networks, in order to predict potential roles of these networks in HTS pathogenesis. The results of GO annotation include terms about the biological process (BP), cellular component (CC), and molecular function (MF); outcomes of KEGG analysis encompass pathways involved in a variety of physiological and pathological processes. Adjusted P value < 0.05 was set as the cut-off value for GO and KEGG enrichment analysis.

### Statistical analysis

Statistical analysis was carried out using GraphPad Prism (version 9.0.1). Expression levels of miRNAs, lncRNAs, and genes between HTS and control groups were presented as mean ± SD. Student’s t-test and Mann–Whitney test were performed for data with equal and unequal variances, respectively. P < 0.05 was considered to reach statistical significance.

## Results

### miR-422a, miR-2116-3p, and miR-3187-3p are downregulated in HTS tissues and fibroblasts

**Fig. 2:**
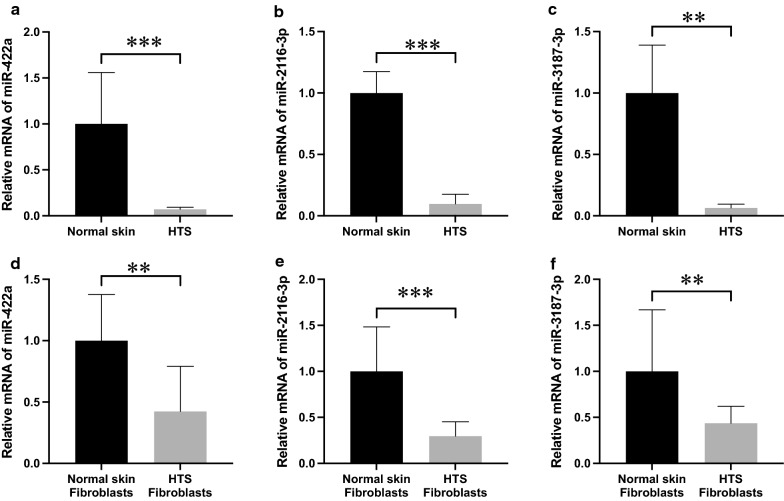
Expression of miR-422a, miR-2116-3p, and miR-3187-3p between normal skin and HTS. **a**–**c** In HTS tissues, the three miRNAs were downregulated. **d**–**f** In HTS-derived fibroblasts, expression levels of the three miRNAs were significantly lower. Results were shown as mean ± SD (n = 10, **P < 0.01, ***P < 0.001). HTS, hypertrophic scar

To compare expression levels of miR-422a, miR-2116-3p, and miR-3187-3p between HTS and normal skin, 10 HTS tissues as well as normal skin samples were subject to qRT-PCR. The results revealed that each expression of these three miRNAs was significantly lower in HTS tissues as opposed to normal skin samples (Fig. 2a–c). To further confirm the expression pattern, qRT-PCR was employed to dissect mRNA levels of the three miRNAs in fibroblasts derived from HTS and normal skin tissues. The outcomes were consistent with those in tissues and indicated that all of the three miRNAs were downregulated in HTS fibroblasts (Fig. 2d–f). Taken together, these findings suggest that miR-422a, miR-2116-3p, and miR-3187-3p may have a close relationship with HTS formation, and play a protective role in the process.

### Construction of ceRNA networks

**Fig. 3:**
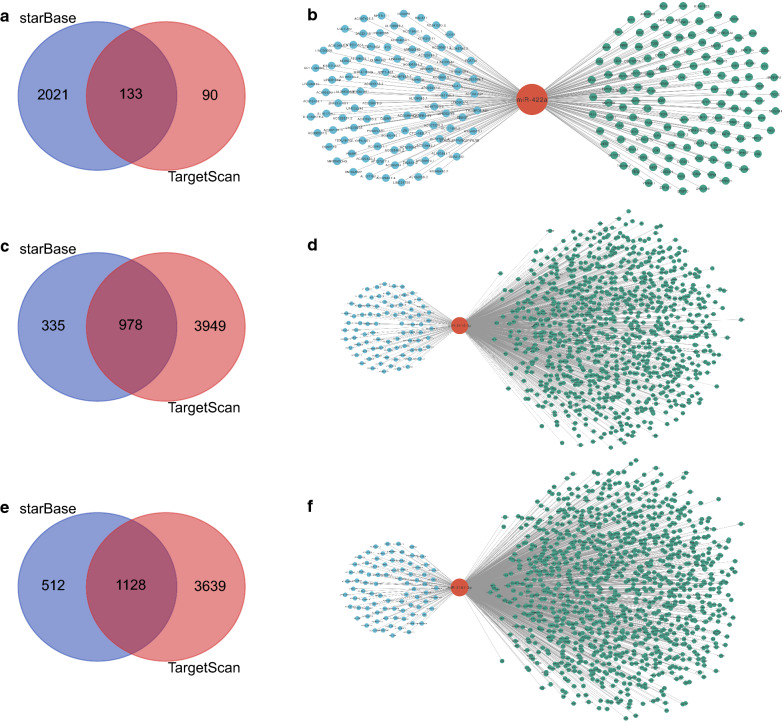
Target genes of miR-422a (**a**), miR-2116-3p (**c**), and miR-3187-3p (**e**) were acquired by overlapping the results of starBase and TargetScan. A ceRNA network was constructed for each of miR-422a (**b**), miR-2116-3p (**d**), and miR-3187-3p (**f**)

Given that in the pathogenesis of most diseases including HTS, ceRNA networks are the major functional pattern of miRNAs, both upstream lncRNAs and downstream genes were predicted for each of miR-422a, miR-2116-3p, and miR-3187-3p to construct ceRNA networks. As for miR-422a, a total of 101 target lncRNAs were obtained using the starBase database; 133 target genes were acquired after overlapping data from starBase and TargetScan (Fig. 3a). Using these lncRNAs and mRNAs, a ceRNA network centering around miR-422a was constructed (Fig. 3b). In identical ways, by overlapping results from starBase and TargetScan databases (Fig. 3c), the ceRNA network for miR-2116-3p was established with 85 lncRNAs and 978 target genes (Fig. 3d). Similarly, concerning miR-3187-3p, 84 target lncRNAs and 1128 genes were predicted through starBase and intersection of outcomes in starBase as well as TargetScan, respectively (Fig. 3e). The lncRNAs and target genes were then utilized to set up a ceRNA network of miR-3187-3p (Fig. 3f).

### Establishment of PPI networks and identification of hub genes

**Fig. 4:**
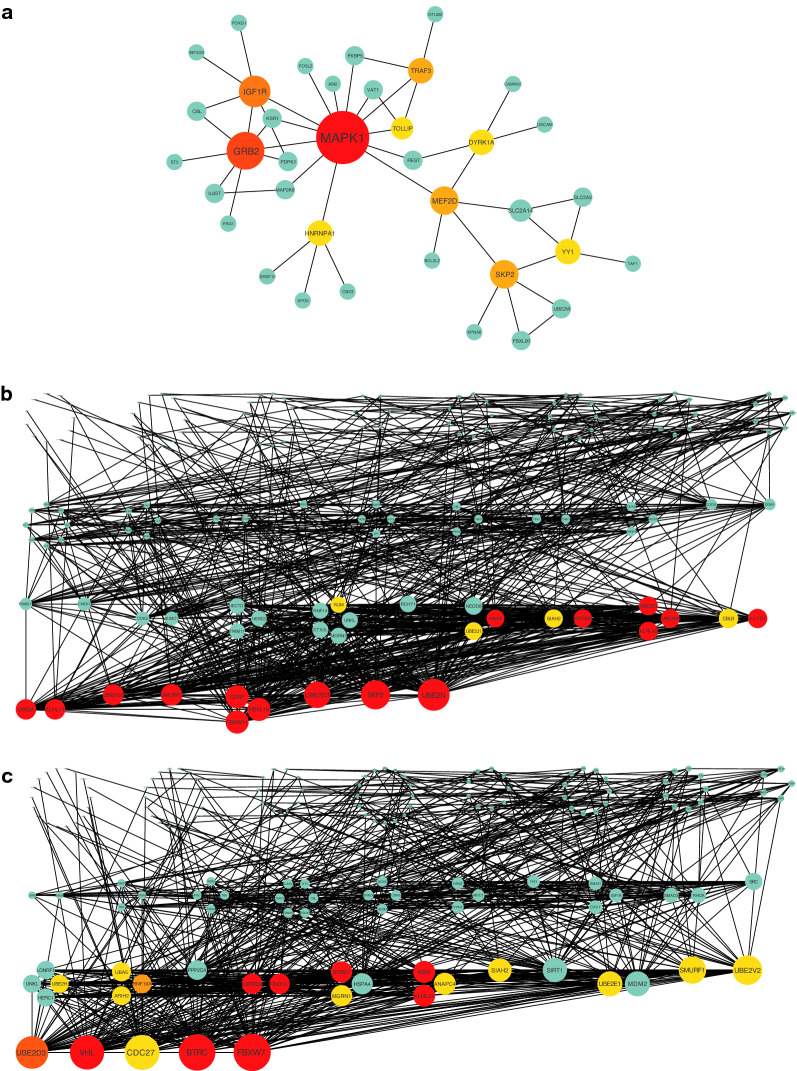
PPI networks of miR-422a (**a**), miR-2116-3p (**b**), and miR-3187-3p (**c**), with top 10, 20, and 20 hub genes (highlighted), respectively. The node size increased in proportion to its degree, which was calculated by the built-in algorithm in Cytoscape. PPI, protein–protein interaction

In light of the fact that miRNAs tend to regulate HTS development through interacting with mRNAs of protein-coding genes, target genes in each of the three ceRNA networks were utilized to build up a distinct PPI network in order to find hub genes. The PPI network of miR-422a encompassed 87 edges and 133 nodes, and analysis of this network detected 10 hub genes (Fig. 4a). In terms of miR-2116-3p, its PPI network consisted of 3926 edges and 975 nodes, 20 of which were shown to be hub genes (Fig. 4b). Concerning miR-3187-3p, the PPI network involved 5047 edges and 1125 nodes, the analysis of which identified 20 hub genes (Fig. 4c).

### GO and KEGG enrichment analysis

**Fig. 5:**
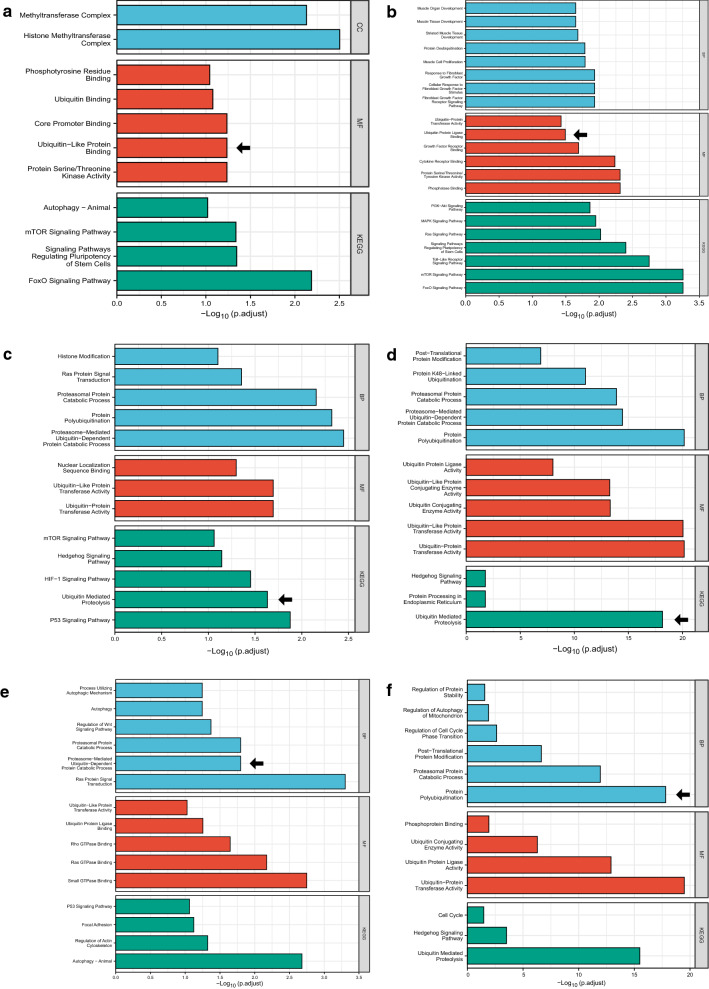
GO and KEGG analysis of miR-422a (**a**) with its hub genes (**b**), miR-2116-3p (**c**) as well as its hub genes (**d**), and miR-3187-3p (**e**) together with its hub genes (**f**) were all related to ubiquitination (arrow). GO annotations include 3 categories: biological process (BP), cellular component (CC), and molecular function (MF). *GO* Gene Oncology, *KEGG* Kyoto Encyclopedia of Genes and Genomes

To gain insights into potential roles of miR-422a, miR-2116-3p, and miR-3187-3p, GO and KEGG enrichment analyses were conducted for both target and hub genes in the ceRNA networks. For the miR-422a-centered network, target and hub genes were both closely related with ubiquitin binding, PI3K/Akt/mTOR signaling pathway, and signaling pathways regulating pluripotency of stem cells (Fig. 5a, b). As to the network centering miR-2116-3p, target and hub genes were consistently enriched in proteasome-mediated ubiquitin-dependent protein catabolic process, ubiquitin-protein transferase activity, and ubiquitin-mediated proteolysis (Fig. 5c, d). Regarding miR-3187-3p, analysis of its network demonstrated that functions of target genes were similar to those of hub genes, focusing on proteasomal protein catabolic process as well as ubiquitin-protein ligase activity (Fig. 5e, f). Taken together, these findings highlight that the three miRNAs may be associated with ubiquitination during HTS formation.

### Integrated analysis of a three-miRNA ceRNA network

**Fig. 6:**
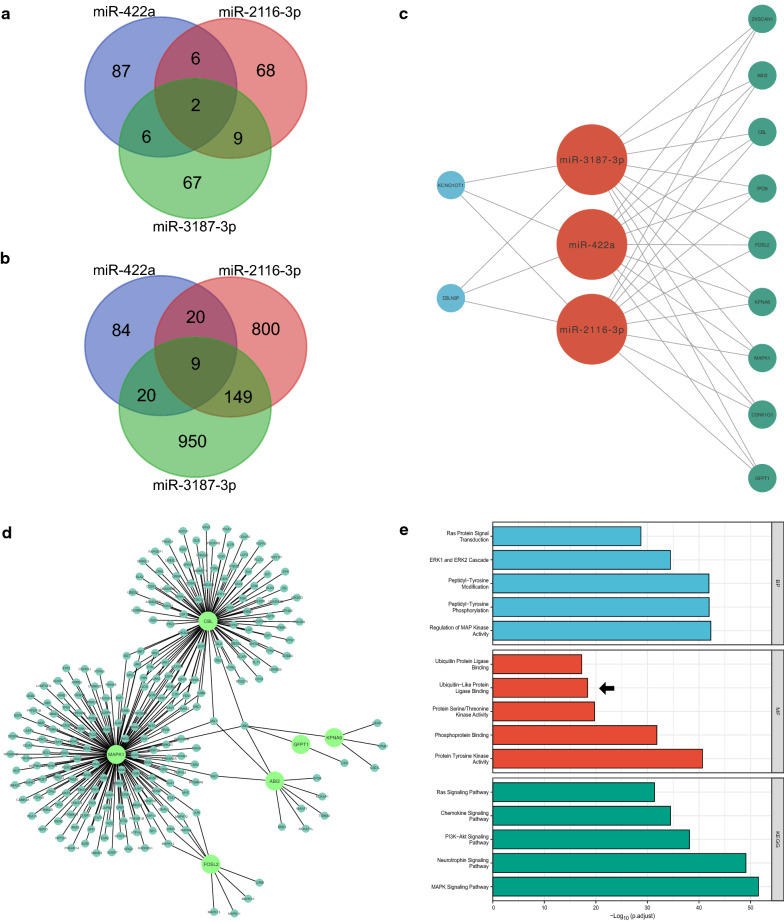
Establishment and functional enrichment analysis of the three-miRNA ceRNA network. **a** Two mutual lncRNAs were required through overlapping target lncRNAs of the three miRNAs. **b** Nine mutual genes were the overlap of target genes of the three miRNAs. **c** The three-miRNA ceRNA network centering around miR-422a, miR-2116-3p, and miR-3187-3p. **d** The PPI network of 9 mutual genes and their interacting proteins, among which 6 hub genes were highlighted. **e** GO and KEGG analysis of 9 mutual genes and their interacting proteins in the three-miRNA network were related to ubiquitination (arrow). GO annotations include 3 categories: biological process (BP), cellular component (CC), and molecular function (MF). *GO* Gene Oncology, *KEGG* Kyoto Encyclopedia of Genes and Genomes

In an effort to develop a comprehensive ceRNA network based on miR-422a, miR-2116-3p, and miR-3187-3p, their target lncRNAs and genes were intersected to reap 2 mutual lncRNAs (EBLN3P and KCNQ1OT1) (Fig. 6a) and 9 mutual genes (IPO9, ABI2, ZKSCAN1, FOSL2, GFPT1, MAPK1, CBL, CSNK1G1, KPNA6) (Fig. 6b), respectively. The resultant three-miRNA ceRNA network was demonstrated as the diagram (Fig. 6c). To explore its potential roles in HTS pathogenesis, the 9 mutual genes were subject to NetworkAnalyst (http://www.networkanalyst.ca/) to determine their interacting proteins, which were used to build up a PPI network (Fig. 6d) and analyze related GO terms as well as KEGG pathways (Fig. 6e). The enrichment analysis result implied that these genes interacting with the network may be significantly connected to MAPK signaling pathway and ubiquitin-protein ligase binding, which also existed in the outcomes of the ceRNA RNA networks of miR-422a, miR-2116-3p, and miR-3187-3p. Overall, these findings suggest that the three miRNAs may synergistically engage in HTS development through regulating the same genes and form a ceRNA network associated with ubiquitination.

### Validation of the three-miRNA ceRNA network

**Fig. 7:**
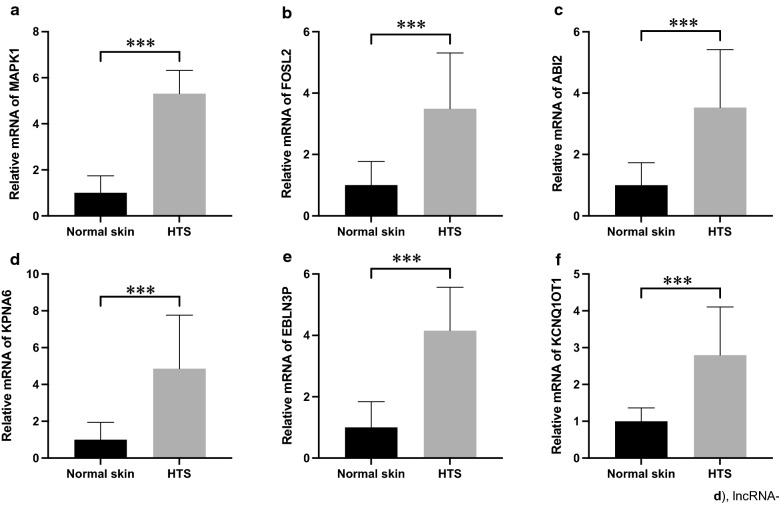
Validation of selected genes and lncRNAs in the three-miRNA ceRNA network. MAPK1 (**a**), FOSL2 (**b**), ABI2 (**c**), KPNA6 (**d**), lncRNA-EBLN3P (**e**), and lncRNA-KCNQ1OT1 (**f**) were significantly overexpressed in HTS tissues. Results were shown as mean ± SD (n = 10, ***P < 0.001). *HTS* hypertrophic scar

**Fig. 8:**
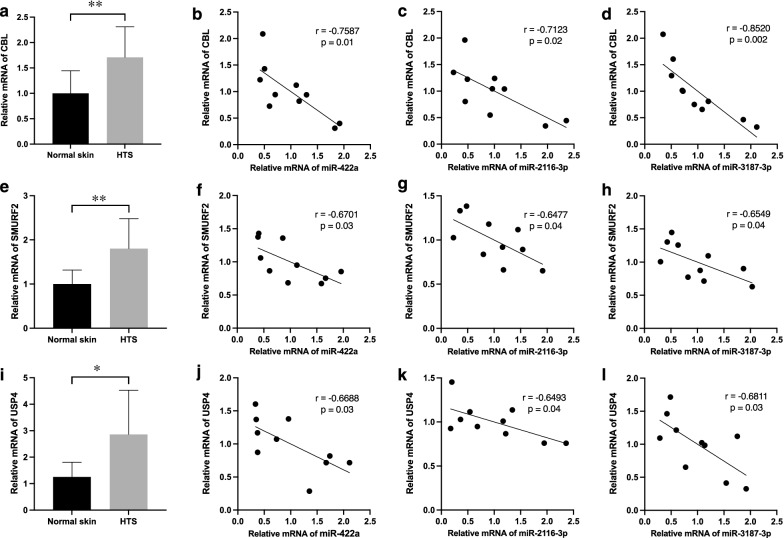
Validation of three ubiquitination-related genes in HTS tissues. CBL (**a**–**d**), SMURF2 (**e**–**h**), and USP4 (**i**–**l**) were upregulated in HTS tissues and negatively related to expression levels of miR-422a, miR-2116-3p, and miR-3187-3p. Results were shown as mean ± SD (n = 10, *P < 0.05, **P < 0.01). *HTS* hypertrophic scar

To further confirm our hypothesis, expression levels of some lncRNAs and genes in the three-miRNA network were detected in HTS tissues and normal skin samples. The qRT-PCR results showed that 2 lncRNAs (KCNQ1OT1 and EBLN3P) and 4 protein-coding genes (MAPK1, FOSL2, ABI2, and KPNA6) were significantly overexpressed in HTS (Fig. 7). Furthermore, three ubiquitination-related genes (CBL, SMURF2, and USP4) were validated to be upregulated and negatively related to the expression levels of miR-422a, miR-2116-3p, and miR-3187-3p (Fig. 8). Collectively, these data supported the notion that the three-miRNA ceRNA network may participate in HTS formation and correlate with ubiquitination.

## Discussion

Hypertrophic scars are a kind of skin fibrotic disorder and may have repercussions on the quality of life for patients, but the underlying mechanism remains poorly understood [13]. During the process of HTS formation, miRNAs have been observed to play a regulatory role [14]. A preliminary microarray test on HTS tissues has identified top 40 up and downregulated miRNAs, among which miR-422a, miR-2116-3p, and miR-3187-3p displayed relatively great variations between HTS and control samples. Moreover, according to the prediction of starBase and TargetScan databases, the three miRNAs may target molecules that have been confirmed to play an important role in hypertrophic scarring. For example, miR-422a could target MAPK1 which is a key component of the MAPK signaling pathway, which is actively involved in HTS formation; miR-2116-3p might bond with either COL1A1 whose upregulation causes excessive collagen deposition in HTS, or SERPINE1 that is also known as PAI-1 and has proven a fibrotic gene related to HTS [15]; miR-3187-3p was predicted to inhibit IGF1R whose deficiency has been shown to be significantly associated with the fibrosis of skin [16]. These data imply that miR-422a, miR-2116-3p, and miR-3187-3p may be downregulated and take part in biological processes during HTS formation. This study utilized qRT-PCR to confirm the expression pattern, where all of these three miRNAs were significantly lower in both tissues and fibroblasts of HTS than those of normal skin. These results suggest that miR-422a, miR-2116-3p, and miR-3187-3p are downregulated and might perform a role during HTS pathogenesis.

CeRNA networks, where lncRNAs and protein-coding genes competitively sponge miRNAs, have been shown to engage in multiple diseases including fibrosis. For example, miR-422a was shown to target MEF2D and be sponged by lncRNA D63785, which promoted chemoresistance in gastric cancer [17]; a LINC01433/miR-2116-3p/MYC positive feedback loop was identified to facilitate breast cancer cell proliferation, migration, and epithelial to mesenchymal transition (EMT) [6]; lncRNA H19 targeting miR-3187-3p/GAB1 axis acted as a ceRNA to promote fibroblast proliferation, migration, and invasion in HTS [7]. However, the potential roles of the miR-422a and miR-2116-3p in HTS formation remain unclear. In this study, for each of miR-422a, miR-2116-3p, and miR-3187-3p, the target lncRNAs and genes were predicted to construct a ceRNA network. In addition, the target genes of each miRNA were utilized to establish a distinct PPI network, the analysis of which identified its hub genes. Both target genes and hub genes of every miRNA were subject to GO and KEGG enrichment analysis, in order to explore possible functions of these networks.

The present results showed that miR-422a was significantly related to PI3K/Akt/mTOR signaling pathway and signaling pathways regulating pluripotency of stem cells, supported by previous studies. For instance, miR-422a was confirmed to inhibit glioma cell proliferation, invasion, and migration by targeting PIK3CA, which is a critical member of PI3K/Akt signal pathway [18]; miR-422a was proven to directly target AKT1 to inhibit PI3K/Akt pathway and colorectal cancer cell proliferation [19]. In addition, miR-422a was shown to weaken breast cancer stem cell proliferation in vitro and in vivo [20]. Although these results were obtained from cancer research, HTS development is closely highly associated with PI3K/Akt/mTOR signaling pathway [21] and can also be influenced through manipulating and stem cells [22]. Therefore, miR-422a may regulate HTS formation by impacting PI3K/Akt/mTOR signaling pathway or stem cells.

In this study, each ceRNA network for miR-422a, miR-2116-3p, and miR-3187-3p was predicted to be significantly related to ubiquitination, which was also one of the significant terms in the enrichment analysis results of the integrated ceRNA network centering around the three miRNAs. By means of qRT-PCR, we verified the negative correlation between the three miRNAs and three ubiquitination-related genes (CBL, SMURF2, and USP4), which are key components of the ubiquitin–proteasome system and have been shown to engage in the ubiquitination and degradation of some pivotal genes implicated in HTS [23, 24]. Consistent with these findings, miR-422a was reported to directly target SKP2 [25], which is an E3 ubiquitin ligase and was confirmed to promote fibroblast proliferation in pulmonary fibrosis through via ubiquitin–proteasome pathway [26]. In addition, miR-2116-3p was shown to interact with and inhibit PIWIL1 [27], and silencing of PIWIL1 was reported to upregulate FBXW7 [28], which is a component of E3 ubiquitin-protein ligase complex and was observed to induce expression of fibrotic genes including fibronectin during pulmonary fibrosis [29]. Also, miR-3187-3p was confirmed to target ASXL1 [30], a histone H2A deubiquitinase [31]. Collectively, these studies raise the possibility that miR-422a, miR-2116-3p, and miR-3187-3p may affect the pathogenesis of HTS by influencing the ubiquitin–proteasome system, which plays a pivotal role in multiple skin fibrosis diseases [32]. However, more direct evidence for complicated mechanisms between the three miRNAs and ubiquitination needs to be found in future research, by means of cell transfection experiments.

In a ceRNA network, a single lncRNA has the potential to simultaneously sponge more than one miRNA. However, this one-to-many pattern has not been investigated in research about the pathogenesis of HTS, and the most of research studying the three miRNAs has only focused on a single miRNA [4]. In this research, a three-miRNA ceRNA network was constructed using the overlapping lncRNAs and target genes of miR-422a, miR-2116-3p, and miR-3187-3p, thereby exploring potential functions that the three miRNAs may perform through regulating the same genes. According to the GO and KEGG functional enrichment analysis, in addition to ubiquitination, this integrated network was predicted to be significantly closed to MAPK signaling pathway, which plays a critical role in HTS pathogenesis. Interestingly, among the three miRNAs, only miR-422a was reported to impact MAPK signaling pathway [19], whose relationship with the other two miRNA is worthy of scholarly attention.

To validate the three-miRNA network in HTS, qRT-PCR was used to determine expression levels of some lncRNAs as well as genes in the network. The results revealed that MAPK1, FOSL2, ABI2, KPNA6, CBL, lncRNA-KCNQ1OT1, and lncRNA-EBLN3P were overexpressed in HTS tissues. MAPK1 was shown to be targeted by miR-422a [19] and a key component of MAPK signaling pathway, which is actively involved in HTS formation. FOSL2, also known as FRA-2, is an activator protein 1 (AP-1) transcription factor and was reported to promote pulmonary fibrosis [33], cardiac fibrosis [34], and skin fibrosis [35], and be regulated by lncRNA UCA1 in ceRNA networks during tumorigenesis [36, 37]. LncRNA KCNQ1OT1 silencing in vivo was confirmed to alleviate cardiac fibrosis by inhibiting the TGF-β1 signaling pathway [38]. These findings suggest that MAPK1, FOSL2, and lncRNA KCNQ1OT1 have the potential to take part in HTS pathogenesis through interacting with miR-422a, miR-2116-3p, and miR-3187-3p. However, there is a shortage of research on the roles of ABI2, KPNA6, CBL, and lncRNA EBLN3P in fibrotic disorders such as HTS; relationships between these genes and the three miRNAs still need further investigation by experiments.

## Conclusions

In conclusion, this study showed that miR-422a, miR-2116-3p, and miR-3187-3p are downregulated in HTS tissues and fibroblasts. Furthermore, a distinct ceRNA network for each of the three miRNAs and a three-miRNA (miR-422a, miR-2116-3p, and miR-3187-3p) ceRNA network were proposed and analyzed using bioinformatics analysis, which implied that these networks were significantly related to ubiquitination. Moreover, the expression of the three-miRNA ceRNA network and its correlation with ubiquitination-related genes were validated by qRT-PCR. Overall, the current research suggests that miR-422a, miR-2116-3p, and miR-3187-3p may play a role in HTS formation and correlate with ubiquitination.

## Data Availability

The microarray data of miR-422a, miR-2116-3p, and miR-3187-3p in hypertrophic scars are openly available at https://doi.org/10.3892/mmr.2017.6104.

## Abbreviations

ceRNA: Competitive endogenous RNA
HTS: Hypertrophic scar
(qRT‑PCR): Quantitative real‑time PCR
miRNA: MicroRNA
lncRNA: Long non-coding RNA
PPI: Protein–protein network
GO: Gene Oncology
KEGG: Kyoto Encyclopedia of Genes and Genomes
STRING: Search Tool for the Retrieval of Interacting Genes
MCC: Maximal clique centrality
EBLN3P: Endogenous bornavirus-like nucleoprotein
KCNQ1OT1: Potassium voltage-gated channel subfamily Q member 1 opposite strand 1
IPO9: Importin-9
ABI2: Abl interactor 2
ZKSCAN1: Zinc finger protein with KRAB and SCAN domains 1
FOSL2: Fos-related antigen 2
GFPT1: Glutamine–fructose-6-phosphate aminotransferase 1
MAPK1: Mitogen-activated protein kinase 1
CBL: E3 ubiquitin-protein ligase casitas B lymphoma
CSNK1G1: Casein kinase I isoform gamma-1
KPNA6: Importin subunit alpha-7
EMT: Epithelial to mesenchymal transition
MEF2D: Myocyte enhancer factor-2D
PIK3CA: Phosphatidylinositol 4,5-bisphosphate 3-kinase catalytic subunit alpha isoform
AKT1: RAC-alpha serine/threonine-protein kinase
SKP2: S-phase kinase-associated protein 2
PIWIL1: Piwi-like protein 1
FBXW7: F-box/WD repeat-containing protein 7
ASXL1: Polycomb group protein
