# Correction to: Identification and functional analysis of a three-miRNA ceRNA network in hypertrophic scars

**DOI:** 10.1186/s12967-022-03241-w

**Published:** 2022-01-18

**Authors:** Zewei Zhang, Xin Huang, Jiahao Yang, Shuchen Gu, Yixuan Zhao, Yunhan Liu, Yimin Khoong, Shuqi Wang, Shenying Luo, Tao Zan, Guangshuai Li

**Affiliations:** 1 Department of Plastic and Reconstructive Surgery, The First Affiliated Hospital of Zhengzhou University, Zhengzhou, China; 2 Department of Plastic and Reconstructive Surgery, Shanghai Ninth People’s Hospital, Shanghai Jiaotong University School of Medicine, Shanghai, China; 3 Department of Orthopedic, The First Affiliated Hospital of Zhengzhou University, Zhengzhou, China

## Correction to: J Transl Med (2021) 19:451 https://doi.org/10.1186/s12967-021-03091-y

In the original publication [1], there was an incorrect funding section. The incorrect and correct funding information is published in this correction article. The original article has been updated.

**Incorrect funding**

This work was supported by the National Natural Science Foundation of China (Grant Number: 81873538) and Key Scientific Research Projects of Colleges and Universities in Henan Province (Grant Number: 20A320033).

**Correct funding**

This work was supported by the National Natural Science Foundation of China (Grant Number: 81772086, 82072177) and Key Scientific Research Projects of Colleges and Universities in Henan Province (Grant Number: 20A320033).
